# Association Between State Supplemental Nutrition Assistance Program Policies, Child Protective Services Involvement, and Foster Care in the US, 2004-2016

**DOI:** 10.1001/jamanetworkopen.2022.21509

**Published:** 2022-07-13

**Authors:** Michelle Johnson-Motoyama, Donna K. Ginther, Patricia Oslund, Lindsay Jorgenson, Yoonzie Chung, Rebecca Phillips, Oliver W. J. Beer, Starr Davis, Patricia L. Sattler

**Affiliations:** 1 The Ohio State University College of Social Work, Columbus; 2 The University of Kansas Institute for Policy and Social Research, Lawrence; 3 University of Maryland School of Social Work, Baltimore; 4 The University of Kansas School of Social Welfare, Lawrence

## Abstract

**Importance:**

Public assistance policies may play a role in preventing child maltreatment by improving household resources among families of low incomes. The Supplemental Nutrition Assistance Program (SNAP) is one of the largest public assistance programs in the US. However, the association of state SNAP policy options to Child Protective Services (CPS) outcomes has not been rigorously examined.

**Objective:**

To model the association of state SNAP policies with changes in CPS and foster care outcomes in the US over time.

**Design, Setting, and Participants:**

This cohort study used panel data to examine the association between SNAP policy options and study outcomes from 2004 to 2016 for 50 US states and the District of Columbia in 2-way fixed-effects regression models. The count of SNAP policies was used as an instrument for SNAP caseloads in instrumental variables models. Data analysis was conducted in November 2021.

**Exposures:**

The adoption of 1 or more state SNAP income generosity policies that improves or stabilizes household resources for SNAP participants.

**Main Outcomes and Measures:**

Reports of child maltreatment accepted for CPS investigation, children in substantiated reports, and children receiving foster care services for all forms of maltreatment, and specifically for child neglect per 100 000 child population.

**Results:**

The mean (SD) number of SNAP income generosity policies increased from 1.47 (0.95) in 2004 to 2.37 (0.94) in 2010, to 2.49 (0.86) in 2016 across states; the median increased from 1 to 3 (range, 0-4) over the same period. A count of state income generosity policies was associated with large reductions in reports accepted for CPS investigation (–352.6 per 100 000 children; 95% CI, –557.1 to –148.2). Income generosity policy was associated with –94.8 (95% CI, –155.6 to –34.0) fewer substantiated reports and –77.0 (95% CI, –125.4 to –28.6) fewer reports substantiated for neglect per 100 000. Each additional income generosity policy adopted by a state was associated with –45.1 (95% CI, –71.6 to –18.5) to –42.3 (95% CI, –64.8 to –19.8) fewer total foster care placements per 100 000 children.

**Conclusions and Relevance:**

State SNAP policies that improve and stabilize household resources appear to be associated with reductions in CPS involvement and use of foster care. The number of policies implemented had cumulative outcomes beyond individual policy outcomes.

## Key Points

**Question:**

Are state Supplemental Nutrition Assistance Program (SNAP) policy options associated with rates of Child Protective Services involvement and use of foster care services in the US?

**Findings:**

This cohort study including all 50 states and the District of Columbia noted that adoption of SNAP policies increased from 2004 to 2016 and, accompanying the increases, substantiated reports of childhood neglect decreased. In instrumental variables models, policies to operate through SNAP caseloads were identified.

**Meaning:**

The findings of this study suggest SNAP policy options that increase the generosity and stability of household resources may yield valuable population health returns by preventing child maltreatment and the need for costly child welfare interventions.

## Teaser

This cohort study assesses the association of state Supplemental Nutrition Assistance Program expansion with use of child protective services and foster care.

## Introduction

Approximately 37.4% of US children experience a Child Protective Services (CPS) investigation in response to a referral for child maltreatment by their 18th birthday,^1^ and more than 250 000 children enter foster care each year.^2^ Children in households with low and/or unstable incomes experience heightened risk for child maltreatment compared with children in families with greater incomes and stable household resources.^3,4,5^ Observational research suggests public assistance and tax policies may play a role in preventing child maltreatment and foster care placements by improving household resources among families of low income.^6,7,8^ However, to our knowledge, the association of state Supplemental Nutrition Assistance Program (SNAP) policies with child maltreatment outcomes has yet to be examined.

SNAP is a legal entitlement program that offsets the costs of food for families who meet eligibility requirements and is one of the most frequently accessed public assistance programs. Eligible families typically have incomes at or below the poverty line after program deductions and limited assets.^9^ The US Department of Agriculture administers SNAP in cooperation with states. Despite uniform eligibility requirements and benefits, the passage of the Personal Responsibility and Work Opportunity Reconciliation Act of 1996 granted states discretion in program administration through statutes, regulations, and waivers that the 2002 Farm Bill expanded.^10^ These policy options have created considerable variability across states in program access, income generosity, and the maintenance of benefits.^11^

In addition to broader macroeconomic factors, state SNAP policy options have been found to affect SNAP caseloads and program use.^12,13,14,15,16^ For example, broad-based categorical eligibility (BBCE), a policy that waives the SNAP asset test and increases income limits for many households, has been reported to increase caseloads in multiple studies.^13,14,15,16,17^ The exemptions of vehicles from SNAP asset tests,^13,14,17^ the easing of reporting requirements,^14,16^ and the provision of transitional benefits to households exiting the Temporary Assistance to Needy Families (TANF) program^14^ have also been associated with increased caseloads.^14^ Conversely, short recertification periods^13,14,18,19,20^ and biometric requirements (eg, fingerprinting) have been reported to reduce caseloads.^14,16,17,21^

In addition to SNAP caseloads, SNAP policy options may influence CPS caseload dynamics given the effects on household resources for parenting. Variation in the timing of the states’ adoption of SNAP policies provides a natural means to test this hypothesis. Using state panel data, we examined the association of state SNAP policy options that improve and provide stability to household resources to CPS and foster care caseloads from 2004 to 2016. We refer to this subset of state SNAP policy options as *income generosity* policies that (1) increase the gross income limit for applicants under BBCE, (2) exclude legally obligated child support payments from the payer’s total income, (3) provide transitional SNAP benefits to families leaving TANF or state-funded cash assistance programs, and (4) use the simplified reporting option, which reduced requirements for reporting changes in household circumstances. We examined the association between the count of income generosity policies and our study outcomes. We hypothesized that income generosity policies would increase SNAP caseloads and reduce CPS and foster care outcomes.

## Methods

### Study Design

We created a state longitudinal data panel from data sources to examine the association of income generosity policies with study outcomes and SNAP caseloads from 2004 to 2016 for all 50 states and the District of Columbia (n = 663 state-year pairs). This cohort study was approved by the University of Kansas institutional review board and followed the Strengthening the Reporting of Observational Studies in Epidemiology (STROBE) reporting guideline.

### Measures

#### Independent Variables

**Figure 1.**
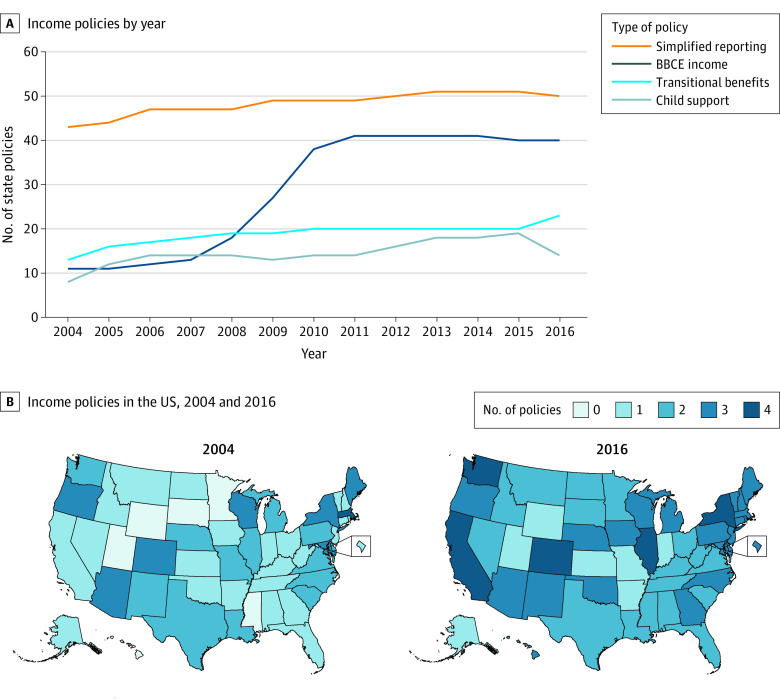
Number and Type of Income Generosity Policies by Year, 2004 – 2016 State income policies by year (A) and US income policies at the start and end of the study (B). BBCE indicates broad-based categorical eligibility.

Income generosity variables were drawn from the SNAP Policy Database^22^ and the SNAP State Options Reports^23^ for all 50 states and the District of Columbia (Figure 1), including use of the BBCE to increase or eliminate the asset test and increase the gross income limit for most SNAP applicants (BBCE), treatment of legally obligated child support payments made to nonhousehold members as an income exclusion rather than a deduction (child support exclusion), transitional SNAP benefits for families leaving TANF or state-funded cash assistance programs (transitional SNAP), and simplified reporting to reduce requirements for reporting changes in household circumstances (simplified reporting). We followed the design of previous research^15^ to create a summary count of income generosity policies in a state over time (income generosity) that accounts for potential measurement error in the year that a state implemented each policy and the fact that states often adopt multiple policies in the same year, making it difficult to disentangle the outcomes of a single policy. Whereas others^15^ used the average number of policies, we used the total count because our analysis focused on a subset of all state-controlled SNAP policies.

#### Dependent Variables

We examined the outcomes of state income generosity policies associated with CPS and foster care caseloads using data drawn from the National Child Abuse and Neglect Data System (NCANDS) Child File and the Adoption and Foster Care Reporting and Analysis System (AFCARS), a federal data collection initiative that collects case-level information from all state and tribal Title IV-E agencies for all children in foster care, from 2004 to 2016.^24,25^ The NCANDS measures included reported incidents of child maltreatment accepted for investigation (reports), reports substantiated for child maltreatment (substantiation), and children receiving foster care services (foster care) by state and year. Most children come to the attention of CPS for reasons of neglect (74.9%)^26^ or omissions in care.^27^ Therefore, we examined NCANDS outcomes for all forms of child maltreatment and neglect specifically given the importance of household resources in caregiving. In NCANDS, children receiving foster care services have an accepted report of child maltreatment; however, several state-year pairs were missing for foster care from NCANDS in our panel. Therefore, we included a measure of foster care caseloads from AFCARS. As many as 35% of children recorded in AFCARS may enter foster care for reasons other than child maltreatment,^28^ so we used both NCANDS and AFCARS data to enhance study validity. All outcomes were converted to rates per 100 000 of the child population. We measured SNAP caseloads using data from the University of Kentucky Center for Poverty Research National Welfare Data.^29^ eTable 1 in the Supplement provides details on all data sources and missing data.

#### Control Variables

Control variables were gathered from the Annual Social and Economic Supplement of the Current Population Survey (ASEC)^30^ and the University of Kentucky National Welfare Data^29^ and include the presence of refundable state earned income tax credit programs; the log of the real state minimum wage; state unemployment rates; share living in cities; the log of real personal income; and child population by age. In addition, race and ethnicity and nativity have been associated with disparities in study outcomes in past research; therefore, we controlled for share of immigrants, Asian persons, non-Hispanic Black persons, persons of other races, and share of Hispanic persons of any race following census definitions. Differential response is a CPS reform associated with child welfare caseload reductions.^31^ Therefore, we adjusted for the presence of differential response programs and other sources of variation in child welfare system policies and practices between states in all analyses (eMethods in the Supplement). In robustness checks we adjusted for state-funded cash assistance programs and the US opioid epidemic, which have been associated with child welfare caseload dynamics in past studies.^6,32^

### Statistical Analysis

All statistical analysis was performed using Stata, version 16.1 (StataCorp LLC) in November 2021. All hypothesis tests were 2-sided, with statistical significance of *P* < .05. As with previous studies,^15^ we assessed the association between SNAP policies with CPS and foster care outcomes, using the policy count variable (income generosity) and individual policies. We regressed case rates per 100 000 population on reports, substantiated reports, reports substantiated for neglect, overall children receiving foster care services (NCANDS), children in substantiated reports receiving foster care services (NCANDS), children in substantiated reports for reasons of neglect receiving foster care services (NCANDS), total foster care placements (AFCARS), and foster care placements owing to neglect (AFCARS), using a 2-way fixed effects model with controls for state and year-state fixed effects. We also included estimates of each policy in place of the policy count variable.

As a robustness check, we used the income generosity policy count variable as an instrument for SNAP caseloads measured as households with children, using 2-stage least-squares analysis. This approach assumes that SNAP income generosity policies are associated with child outcomes only through SNAP caseloads. The instrumental variables estimate is the local average treatment effect of SNAP income generosity policies on CPS and foster care outcomes in states that decided to adopt those policies. We adjusted the 2-stage least-squares analysis estimates to be analogous to the 2-way fixed-effects estimates. In both sets of estimates, SEs were clustered at the state level. Additional methodologic details appear in the eMethods in the Supplement.

## Results

The mean (SD) number of income generosity policies per state increased from 1.47 (0.95) in 2004 to 2.37 (0.94) in 2010, to 2.49 (0.86) in 2016 during the study period; the median increased from 1 to 3 (range, 0-4) during that same time. Simplified reporting was the policy option most frequently adopted by the end of the study period followed by the BBCE (78%), transitional SNAP (45%), and child support income exclusion (27%). Although some states had waivers to modify SNAP policies before 2002, most began to make changes after the passage of the Farm Bill. Simplified reporting of income became available in 11 states in 2002 and not before. Nine states had waivers to have higher income limits for SNAP eligibility before 2002, and 1 state allowed SNAP transitional benefits for those leaving TANF before 2002. Thus, our measures that start in 2004 capture most of the changes in SNAP income generosity. In 2007, an increasing number of states began implementing the BBCE option to raise the income limits. Changes in the uptake of other policies occurred more steadily over time, with some states opting out of the child support exclusion by the end of the study period. Figure 1B illustrates change over time across states in the adoption of policies.

Table 1 presents descriptive statistics for CPS and foster care caseloads per 100 000 child population in 2004, 2010, and 2016. Reports accepted for investigation increased nationally during the study period, as did reports substantiated for neglect and children with CPS reports who received foster care services (NCANDS). The number of children reported in the AFCARS decreased from 2004 to 2010, then increased between 2010 and 2016 for all foster care placement reasons and specifically for neglect. eFigure 1 in the Supplement shows the geographic variation in study outcomes over time, with some states experiencing decreases in CPS and foster care outcomes and others experiencing considerable increases. Table 2 presents descriptive statistics for covariates used in the analysis for 2004, 2010, and 2016. States added refundable earned income tax credits and increased the use of differential response programs. Real personal income was stagnant during this time, while TANF caseloads fell. Opioid-associated death rates were almost 3 times higher in 2016 than in 2004.

**Table 1.** CPS and Foster Care Outcome Variables for 2004, 2010, and 2016

Variable	Mean (SD)		
	2004	2010	2016
Reports per 100 000 population^a^	3907.51 (1681.40)	4179.12 (1973.35)	4870.47 (2304.52)
Children with substantiated reports per 100 000 population^a^	1024.34 (551.61)	906.91 (507.51)	968.37 (504.83)
Children with substantiated reports for neglect per 100 000 population^b^	647.93 (429.27)	637.82 (430.38)	696.45 (479.24)
Total foster care			
NCANDS per 100 000 population^c^	281.75 (141.80)	292.73 (160.20)	335.20 (219.09)
Children with substantiated reports in NCANDS per 100 000 population^c^	212.13 (115.22)	203.87 (110.67)	236.55 (146.03)
Children with substantiated reports for neglect in NCANDS per 100 000 population^c^	160.59 (94.35)	166.10 (99.01)	197.02 (142.34)
AFCARS per 100 000 population	475.46 (190.06)	411.29 (164.72)	447.42 (204.98)
Children with substantiated reports for neglect foster in AFCARS per 100 000 population	225.04 (110.98)	216.05 (112.95)	263.76 (140.40)

Abbreviations: AFCARS, Adoption and Foster Care Reporting and Analysis System; CPS, Child Protective Services; NCANDS, National Child Abuse and Neglect Data System.

^a^ Missing Alabama, Alaska, Georgia, North Dakota, Oregon, Wisconsin in 2004; Oregon in 2010.

^b^ Missing Alabama, Alaska, Georgia, North Dakota, Oregon, Wisconsin, and Pennsylvania in 2004; Oregon and Pennsylvania in 2010; Pennsylvania in 2016.

^c^ Missing Alabama, Alaska, Georgia, Michigan, New York, North Carolina, North Dakota, Oregon, Pennsylvania, and Wisconsin in 2004; Georgia, North Carolina, New York, and Pennsylvania in 2010; North Carolina, New York, and Pennsylvania in 2016.

**Table 2.** Descriptive Statistics of Covariates for 2004, 2010, and 2016

Covariates	Mean (SD)		
	2004	2010	2016
Refundable state EITC (1 = yes)	0.25 (0.44)	0.41 (0.50)	0.41 (0.50)
Log real state minimum wage	1.85 (0.16)	2.03 (0.09)	2.04 (0.15)
State uses alternative response	0.16 (0.37)	0.25 (0.44)	0.47 (0.50)
Screen out increased 8%	0.88 (0.33)	0.22 (0.42)	0.14 (0.35)
Screen out counts missing (1 = yes)	1.00 (0.00)	0.16 (0.37)	0.14 (0.35)
Share of immigrants	0.01 (0.01)	0.01 (0.01)	0.01 (0.01)
Unemployment rate	5.22 (1.04)	8.76 (2.04)	4.67 (1.00)
Share living in city	0.53 (0.29)	0.54 (0.29)	0.58 (0.30)
Share Black, Non-Hispanic	0.14 (0.14)	0.13 (0.13)	0.13 (0.12)
Share Asian, Non-Hispanic	0.03 (0.06)	0.04 (0.06)	0.04 (0.06)
Share other race, Non-Hispanic	0.05 (0.06)	0.07 (0.07)	0.07 (0.07)
Share Hispanic, any race	0.12 (0.12)	0.15 (0.13)	0.16 (0.13)
Log real personal income	18.73 (1.06)	18.84 (1.04)	18.99 (1.05)
Share of children aged 3-4 y	0.11 (0.01)	0.11 (0.01)	0.11 (0.02)
Share of children aged 5-13 y	0.49 (0.02)	0.49 (0.02)	0.50 (0.02)
Share of children aged 14-17 y	0.24 (0.02)	0.23 (0.02)	0.23 (0.02)
Log of TANF caseloads	9.86 (1.23)	9.69 (1.25)	9.34 (1.23)
Log of Medicaid caseloads	13.10 (1.12)	13.30 (1.11)	13.60 (1.13)
Log of WIC caseloads	11.34 (1.07)	11.49 (1.10)	11.30 (1.12)
Log of free and reduced lunch caseloads	12.75 (1.05)	12.82 (1.07)	12.78 (1.06)
Solely state funded TANF	0.00 (0.00)	0.57 (0.50)	0.61 (0.49)
Opioid deaths per 100 000	5.29 (2.82)	7.61 (3.96)	14.77 (9.18)
Observations, No.	51	51	51

Abbreviations: EITC, earned income tax credit; TANF, Temporary Assistance to Needy Families; WIC, Special Supplemental Nutrition Program for Women, Infants, and Children.

**Figure 2.**
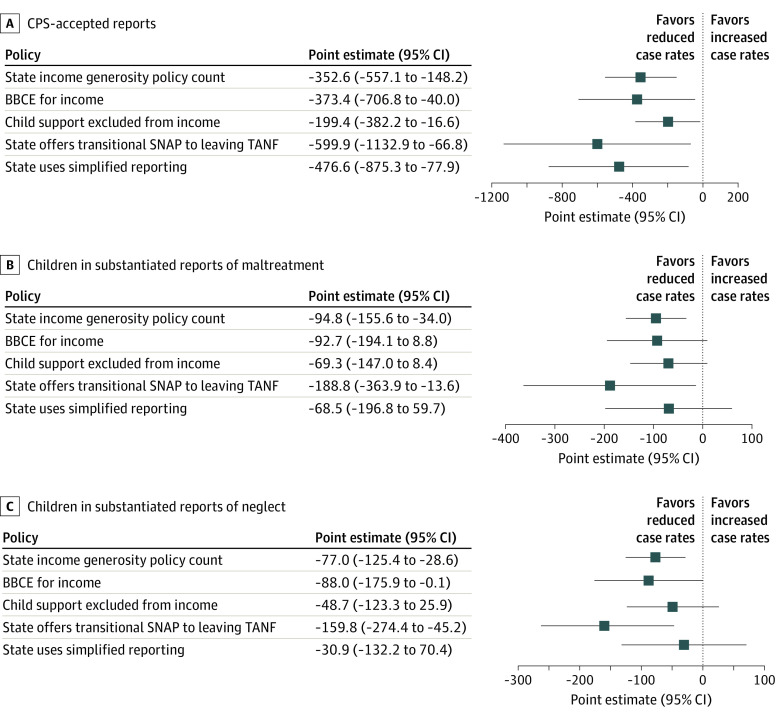
Association of Child Maltreatment per 100 000 Population and Supplemental Nutrition Assistance Program (SNAP) Income Policies A, Reports accepted for Child Protective Services (CPS) investigation. B, Children in substantiated reports of maltreatment. C, Children in substantiated reports for neglect. BBCE indicates broad-based categorical eligibility; TANF, Temporary Assistance to Needy Families.

Figure 2 presents the results of fixed-effects models adjusted for covariates. A state count of income generosity policies was significantly associated with large decreases in reports accepted for CPS investigation of –352.6 (95% CI, –557.1 to –148.2) per 100 000 children. Income policy generosity was associated with –94.8 (95% CI, –155.6 to –34.0) fewer substantiated reports and –77.0 (95% CI, –125.4 to –28.6) fewer reports substantiated for neglect per 100 000 children. Income generosity policies were also associated with fewer foster care placements. With all measured per 100 000 children, each additional income generosity policy adopted by a state was associated with –36.4 (95% CI, –58.1 to –14.7) fewer children with substantiated reports in foster care (NCANDS), –45.1 (95% CI, –71.6 to –18.5) fewer total foster care placements in NCANDS, –42.3 (95% CI, –64.8 to –19.8) fewer total foster care placements in AFCARS, –27.3 (95% CI, –45.1 to –9.5) fewer children with substantiated neglect in foster care (NCANDS), and –27.0 (95% CI, –44.3 to –9.8) fewer children with substantiated neglect reports in foster care placements (AFCARS). The point estimates for foster care placements in NCANDS and AFCARS are similar.

**Figure 3.**
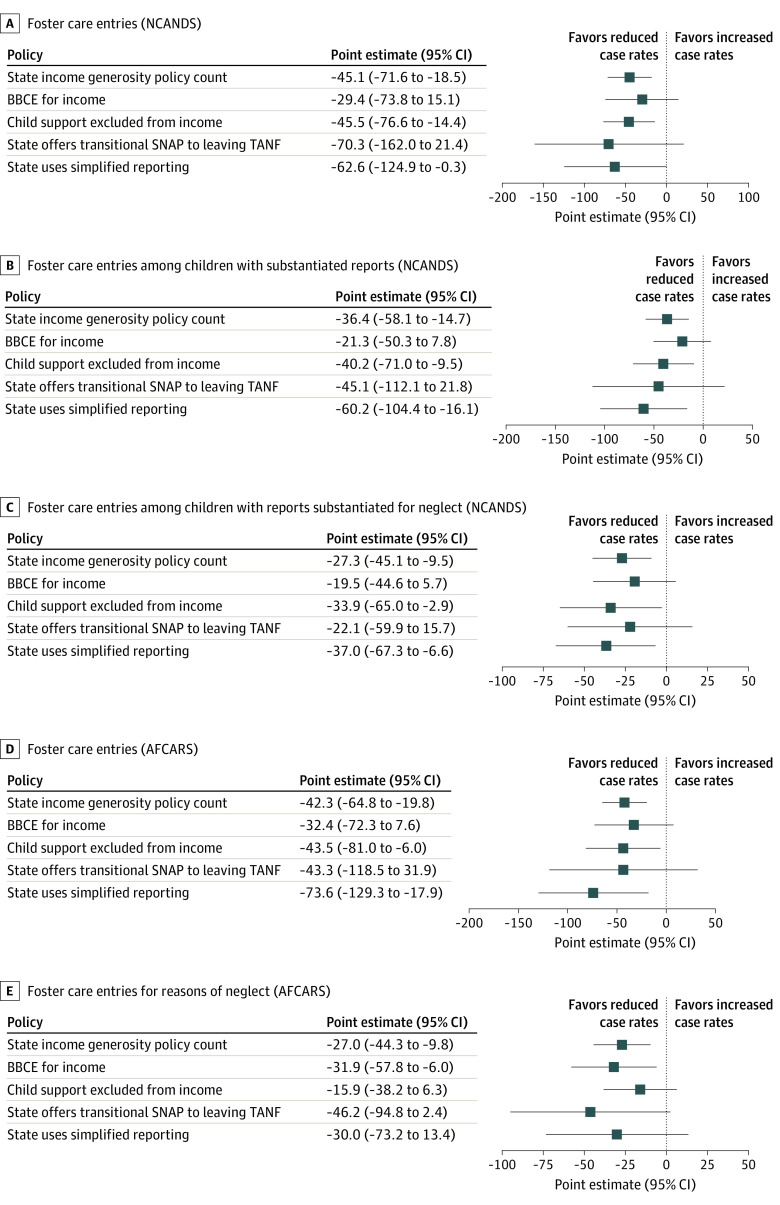
Association of Foster Care Rates per 100 000 Population and Supplemental Nutrition Assistance Program (SNAP) Income Policies A, Foster care entries (National Child Abuse and Neglect Data System [NCANDS]). B, Foster care entries among children with substantiated reports (NCANDS). C, Foster care entries among children with reports substantiated for neglect (NCANDS). D, Foster care entries (Adoption and Foster Care Reporting and Analysis System [AFCARS]). E, Foster care entries for reasons of neglect (AFCARS). BBCE indicates broad-based categorical eligibility; TANF, Temporary Assistance to Needy Families.

Figure 2 and Figure 3 report the association between individual income generosity policies and CPS and foster care outcomes. The BBCE and transitional SNAP benefits were associated with reductions in 3 outcomes. States that implemented transitional SNAP reduced the number of children in substantiated reports per 100 000 children by –188.8 (95% CI, –363.9 to –13.6). States that adopted the BBCE (–88.0; 95% CI, –175.9 to –0.1) or transitional SNAP (–159.8; 95% CI, –274.4 to –45.2) experienced significant decreases per 100 000 children with substantiated reports specifically for reasons of neglect. Increasing income limits through the BBCE reduced foster care placements for reasons of neglect in AFCARS (–31.9; 95% CI, –57.8 to –6.0). A state’s adoption of the child support exclusion or simplified reporting significantly reduced the number of children in all foster care outcomes in NCANDS and AFCARS. This finding suggests that the number of income generosity policies implemented had cumulative associations with CPS and foster care outcomes, beyond the effects of individual policies.

We posited that income generosity policies would increase SNAP caseloads and found each additional income generosity policy increased SNAP caseloads and recipients by 4% to 5% (eTable 2 in the Supplement). Excluding child support from income increased SNAP caseloads as much as 5% to 8%. The use of simplified reporting by states also significantly increased SNAP caseloads by 7% to 11%. However, neither BBCE for income nor transitional benefits for households leaving TANF had a significant association with caseloads. The results in eTable 2 in the Supplement report the first stage of the 2-stage least-squares analysis regression.

As a robustness check we used instrumental variable methods. Each additional income generosity policy was associated with a 5% reduction in SNAP caseloads for families with children (eTable 2 in the Supplement); we therefore multiplied the estimated outcome of caseloads by 5. eFigure 2 in the Supplement compares the estimated outcome of our 2-way fixed-effects estimates from Figure 2 for the income generosity variable with the instrumental variable estimates associated with a 5% reduction in SNAP caseloads. Across all outcomes, the point estimates were similar, with overlapping 95% CIs, and associated with significant decreases in CPS and foster care outcomes. These results suggest that the income generosity policies operate through increasing caseloads, leading to reductions in reports of child maltreatment and use of foster care (eTable 3 in the Supplement).

As a second robustness check, we included additional control variables in our 2-way fixed-effects specifications. First, we added controls for the log of TANF, Medicaid, Special Supplemental Nutrition Program for Women, Infants, and Children, and free and reduced lunch caseloads, and whether a state had a solely state-funded cash assistance program in addition to TANF. Second, we added controls for opioid-associated overdose deaths. As a third set of robustness checks, we included all of these variables in the same specification. eTable 4 in the Supplement reports the robustness checks for income generosity in all 3 specifications: caseloads, the opioid epidemic, and the 2 sets of variables combined. The income generosity variables remained negative and statistically significant for all outcomes.

## Discussion

Using 2 estimation methods, we found states that adopted SNAP income generosity policy options had lower rates of CPS and foster care outcomes for all forms of child maltreatment and specifically for neglect. Our results were robust after controlling for covariates associated with CPS caseload dynamics, such as the opioid epidemic and other public assistance programs. Our finding that income generosity policies operated through SNAP caseloads, which directly influence household resources, provides evidence that SNAP income generosity may influence CPS involvement in low-income households. These SNAP policy options, which increase the generosity and stability of household resources, may yield valuable population health returns by preventing child maltreatment and the need for CPS interventions that are much more costly than SNAP in both human and societal terms. Our findings suggest that increasing access to benefits may reduce CPS and foster care caseloads from 7.6% to 14.3% for every 5% increase in SNAP caseloads. If, indeed, the association of SNAP income generosity to CPS involvement and foster care operates largely through SNAP caseloads as our findings suggest, greater attention to increasing access to SNAP benefits is warranted from a policy perspective.

We observed particularly large estimated reductions in reports and substantiated reports among states offering transitional SNAP benefits to families leaving TANF. In past research, families leaving TANF have been found to be particularly vulnerable to CPS involvement, especially if exits are involuntary and/or unaccompanied by employment.^33,34,35^ Simplified reporting, which stabilizes income, and excluding child support from the calculation of a payer’s income, which incentivizes both SNAP use and payment of child support to single-parent households, were associated with large reductions in reports of maltreatment and foster care. These findings suggest that, in addition to increasing access to SNAP, a policy focus on the stability of household resources is warranted.

### Limitations

This study has limitations. We assume that any changes in CPS caseloads are primarily attributable to SNAP policy changes. Although we used longitudinal data, included a broad range of covariates from a number of reliable sources, and used multiple measures of foster care placement, it is possible that our results may be attributable to unmeasured factors. For example, the SNAP policy option data we could access do not reflect all policy and administrative options that states implemented during the study period. Furthermore, our study period was limited to 2016—the most recent year for which data were available from the US Department of Agriculture Economic Research Service.^22^ Owing to the cohort study design, we cannot infer that SNAP will reduce CPS involvement for every participating household. Neglect estimates must be interpreted cautiously owing to measurement error associated with state definitions and mapping of child abuse and neglect to NCANDS. NCANDS^24^ and AFCARS^25^ are the most reliable sources of longitudinal child maltreatment and foster care data in the US. However, our estimates for child maltreatment are likely conservative given underreporting in state child maltreatment reports.

## Conclusions

Despite its limitations, this study contributes to a growing body of evidence regarding the association between policies that improve and stabilize household resources and rates of CPS involvement.^6,36,37,38^ Research has reported that those who participate in SNAP experience improvements in food security and a range of health, economic, and educational outcomes.^21,39,40,41^ Our findings suggest investments in SNAP may be of even greater value to the health of children than previously known.

## Notes

Supplement. **eMethods.** Detailed Methods
eReferences
**eTable 1.** Data Sources and Information on Missing States/Years **eTable 2.** Association of Income Generosity Policies on SNAP Caseload Measures **eTable 3**. Instrumental Variables Estimates of Association of SNAP Caseloads and Child Maltreatment Outcomes and Foster Care Outcomes **eTable 4.** Robustness Checks for Association of Income Generosity Policies and Child Maltreatment and Foster Care Outcomes **eFigure 1.** Percent Change in Key Indicators From 2004 to 2016, Data per 100 000 Population **eFigure 2.** Association of Child Maltreatment and Foster Care Case Rates per 100 000 Population and SNAP Income Policies—Comparing Fixed Effects and Instrumental Variables Estimates
