# Supplementary material for: Association Between State Supplemental Nutrition Assistance Program Policies, Child Protective Services Involvement, and Foster Care in the US, 2004-2016
Source: JAMA Netw Open. 2022 Jul 13;5(7):e2221509. doi: 10.1001/jamanetworkopen.2022.21509 (PMC9280401; doi:10.1001/jamanetworkopen.2022.21509)
Supplement: Supplement. — eMethods. Detailed Methods eReferences eTable 1. Data Sources and Information on Missing States/Years eTable 2. Association of Income Generosity Policies on SNAP Caseload Measures eTable 3. Instrumental Variables Estimates of Association of SNAP Caseloads and Child Maltreatment Outcomes and Foster Care Outcomes eTable 4. Robustness Checks for Association of Income Generosity Policies and Child Maltreatment and Foster Care Outcomes eFigure 1. Percent Change in Key Indicators From 2004 to 2016, Data per 100 000 Population eFigure 2. Association of Child Maltreatment and Foster Care Case Rates per 100 000 Population and SNAP Income Policies—Comparing Fixed Effects and Instrumental Variables Estimates [file jamanetwopen-e2221509-s001.pdf]

Supplementary Online Content

Johnson-Motoyama M, Ginther DK, Oslund P, et al. Association between State Supplemental Nutrition Assistance Program policies, child protective services involvement, and foster care in the US, 2004-2016. *JAMA Netw Open*. 2022;5(7):e2221509. doi:10.1001/jamanetworkopen.2022.21509

eMethods. Detailed Methods

eTable 1. Data Sources and Information on Missing States/Years

eTable 2. Association of Income Generosity Policies on SNAP Caseload Measures

eTable 3. Instrumental Variables Estimates of Association of SNAP Caseloads and Child Maltreatment Outcomes and Foster Care Outcomes

eTable 4. Robustness Checks for Association of Income Generosity Policies and Child Maltreatment and Foster Care Outcomes

eFigure 1. Percent Change in Key Indicators From 2004 to 2016, Data per 100 000 Population

eFigure 2. Association of Child Maltreatment and Foster Care Case Rates per 100 000 Population and SNAP Income Policies—Comparing Fixed Effects and Instrumental Variables Estimates

This supplementary material has been provided by the authors to give readers additional information about their work.

© 2022 Johnson-Motoyama M et al. *JAMA Network Open.*

eMethods. Detailed Methods

Data Sources and Coding

eTable 1 lists the sources of data for the study and provides details on missing years and states. We used two sources for the SNAP policy variables, the SNAP Policy Database[1] and the SNAP State Options Reports[2] for all 50 states and the District of Columbia. The SNAP Policy Database ended in 2016 thus setting a limit on our analysis sample. We hand-coded the SNAP State Options Reports[2] and discovered discrepancies between policies reported in the tables and those reported in the graphs. In some cases, information on the policy was missing for some years, and we interpolated the missing information as having the same policy as the previous year.

Identifying the effect of SNAP policies is complicated by measurement error and multiple policy changes. As a result, we were guided by previous research by Ganong and Liebman[4] who created averages of SNAP policies. They argue that there may be measurement error in the year that a state implemented a given policy. Like Ganong and Liebman, we also found that states have adopted multiple policies in the same year. Note that Ganong and Liebman used the average number of policies whereas we use the total count since our analysis focused on a subset of all state-controlled SNAP policies. Let

$$PolicyCount_{st} = \sum_{p} Policy_{pst}$$

where *p* is the given policy, *s* is each state and the District of Columbia, and *t* is the year. Exhibit A1 shows the specific policies included in these income generosity and disqualification count measures.

We also analyzed the components of $PolicyCount_{st}$ for each separate state policy. The changes in these variables are listed in Table 1 of the paper. These $PolicyCount_{st}$ variables may

have been adopted at the same time in the same state making it difficult to disentangle the effects of a single policy on outcomes.

We also measured SNAP caseloads from the University of Kentucky Center for Poverty Research National Welfare Data. In order to measure caseloads of SNAP households with children and SNAP households who are single-parent families with children, we applied the percentages of total caseloads of these households reported in *Characteristics of SNAP Households* (3) to the total caseloads in the University of Kentucky data.

In addition to coding SNAP policies, we also used data from the National Child Abuse and Neglect Data System (NCANDS) Child File to create measures of differential response (DR) programs that were implemented and/or halted during the study period. Child protective service (CPS) agencies in approximately half of states provide DR programs to serve children and families with voluntary services to prevent child maltreatment once a report of maltreatment is accepted for investigation. A state was coded as utilizing a DR program if any child was recorded in NCANDS as receiving a CPS determination of alternative response (AR) victim or AR non-victim during the study period. The validity of the state's use of AR designations in NCANDS was triangulated using information from the Quality Improvement Center on Differential Response and state contacts. We controlled for the variation in child welfare policies by adjusting for a 0.5 Standard Deviation change (8% or more) in the proportion of cases that were screened out of a CPS response using data from the NCANDS Child File and the NCANDS Agency File. In some cases, states had missing data on cases screened out and we included an indicator to control for missing data.

We use measures of foster care from both NCANDS and the Adoption and Foster Care Analysis and Reporting System (AFCARS). We use NCANDS foster care data because it is

© 2022 Johnson-Motoyama M et al. *JAMA Network Open.*

closely associated with reports of child abuse and neglect. However, NCANDS has missing data for several states and/or years including Alabama, Alaska, Georgia, New York, North Carolina, North Dakota, Oregon, Pennsylvania and Wisconsin. Our outcome measures of reports, children with substantiated reports, children with reports substantiated for neglect, foster care, children in foster care with substantiated reports, and children in foster care with reports substantiated for neglect are converted to rates per 100,000 of child population. eFigure 1 maps the percent change in child welfare outcomes from 2004 to 2016, data per 100,000 child population. This figure demonstrates the geographic variation in study outcomes over time, with some states experiencing reductions in CPS and foster care outcomes while others experienced considerable increases.

Estimation Methods

All estimates were performed using STATA version 16.1. All hypothesis tests are two-sided tests with statistical significance being p<.05. We use two methods to estimate the impact of state SNAP policies on child maltreatment and foster care outcomes: two-way fixed effects and Instrumental Variables models. The two-way fixed effects approach was used by Ganong and Liebman[4] to estimate the effect of SNAP policy changes on SNAP caseloads and outcomes. Our model is specified as:

$$\Delta log(Caseloads)_{st} = \alpha_s + \beta\Delta PolicyCount_{st} + \gamma\Delta X_{st} + \delta_t + \varepsilon_{st} \qquad (1)$$

where $Caseloads_{st}$ is a measure of SNAP caseloads for state *s* in year *t*. We have four measures of SNAP caseloads: Total caseloads, total recipients, caseloads of households with children, and caseloads of single-parent families with children. These outcomes are a function of state fixed effects (α_s), year fixed effects (δ_t), and additional covariates X_{st} that include the presence of refundable state EITC programs; the log of the real state minimum wage; state share

© 2022 Johnson-Motoyama M et al. *JAMA Network Open.*

of immigrants; state unemployment rates; share living in cities; share of non-Hispanic Blacks, Asians, and other races; share of Hispanics of any race; the log of real personal income; and child population by age. We also include controls for AR response, cases screened out, and the missing screen out variable as described above.

In Figure 3 in the paper, we regress case rates per 100,000 population of the child maltreatment and foster care outcomes (2)

$$\Delta Y_{st} = \alpha_s + \beta \Delta PolicyCount_{st} + \gamma \Delta X_{st} + \delta_t + \varepsilon_{st} \text{ (2)}$$

where Y_{st} is one of eight child maltreatment or foster care measures per 100,000 population of children in the state. The covariates, X_{st} are the same as those used in equation 1. We also included estimates of each separate policy in place of the $PolicyCount_{st}$variable. Essentially, the estimated parameter β is the association between SNAP income generosity policies and child welfare outcomes. We posit that as a state becomes more generous, the magnitude of β will be negative and the number of children with substantiated maltreatment reports or children placed into foster care will decrease. All standard errors are clustered at the state level.

We then estimated a fixed effects instrumental variables model of the association of SNAP caseloads in households with children on our child maltreatment and foster care outcomes using two-stage least squares (TSLS) estimation. This approach assumes that SNAP income generosity policies affects child outcomes only through SNAP caseloads. The instrumental variables estimate is the local average treatment effect of SNAP income generosity policies on CPS and foster care outcomes in states that decided to adopt them. Instrumental variable methods are widely used in economics to correct for this kind of selection bias. An instrument is a variable correlated with SNAP caseloads but uncorrelated with the outcomes of involvement with child protective services and foster care placements. The first stage of the instrumental

© 2022 Johnson-Motoyama M et al. *JAMA Network Open.*

variable model regresses the logarithm of SNAP caseloads on state income generosity policies and is given in equation (3):

$$\Delta log(Caseloads)_{st} = \alpha_s + \lambda \Delta PolicyCount_{st} + \gamma \Delta X_{st} + \delta_t + \varepsilon_{st} \qquad (3)$$

Where $PolicyCount_{st}$ is an exogenous instrument for SNAP caseloads measured as households with children. All covariates are defined as above. The fitted values from this regression are included as a regressor in the second stage regression where CPS and foster care are the outcomes given in equation (4):

$$\Delta Y_{st} = \alpha_s + \phi \Delta \log{(\widehat{Caseloads})_{st}} + \gamma \Delta X_{st} + \delta_t + \varepsilon_{st} \qquad (4)$$

Essentially, $\widehat{Caseloads}$ is a measure of SNAP caseloads that are no longer biased by unobserved factors under the instrumental variables assumptions. All standard errors are clustered at the state level.

Impact of Policies on SNAP Caseloads

We posit that SNAP income generosity policies will increase caseloads. eTable 2 shows the results. We find that each additional income generosity policy increases SNAP caseloads and recipients by 4 to 5 percent. We find that excluding child support from income increases SNAP caseloads as much as 5 to 8%. The use of simplified reporting by states also significantly increases SNAP caseloads by 7 to 11%. Neither BBCE for income nor transitional benefits for households leaving TANF have a significant impact on caseloads. The results in eTable 2 are the first-stage of the TSLS regression.

Robustness Checks

As a first robustness check, we estimated the impact of caseloads on child maltreatment and foster care outcomes where $PolicyCount_{st}$ is an exogenous instrument for SNAP caseloads measured as households with children. The results are presented in eTable 3. We first test for whether we have weak instruments by examining the F-statistic from the first-stage of the two-stage least squares regression. An instrument is considered weak if the F-statistic is < 10.[5] All F-statistics exceed 100.

Since we are regressing maltreatment and foster care case rates on the log of caseloads, the coefficient on log caseloads needs to be transformed. When regressing CPS and foster care outcomes on the logarithm of caseloads, the coefficient is interpreted as a 1% increase in SNAP caseloads is associated with a $\frac{\phi}{100}$ unit decrease in outcomes. Recall from eTable 2 is the first stage of the TSLS estimator. Those results show that each additional SNAP generosity policy increased SNAP caseloads in households with children by 5%. We have transformed the parameter estimates into 1% and 5% estimates of the association of caseloads on child maltreatment and foster care case rates. The reduction in reports, children in substantiated reports, children in reports substantiated for neglect, and foster care placements are very similar using the two methods.

EFigure 2 compares the fixed effects estimates with the instrumental variables estimates that were adjusted for a 5% reduction in SNAP caseloads. Across all outcomes, the point estimates using two-way fixed effects and instrumental variables estimates are remarkably similar, with overlapping confidence intervals, and are associated with significant reductions in CPS and foster care outcomes. These results indicate that the income generosity policies operate through increasing caseloads, leading to reductions in child maltreatment and removals into foster care.

© 2022 Johnson-Motoyama M et al. *JAMA Network Open.*

In order to determine whether our results are robust to other sets of variables, we included controls for the log of TANF caseloads, Medicaid caseloads, WIC caseloads, free and reduced lunch caseloads and whether a state had a solely state-funded cash assistance program in addition to TANF in our two-way fixed effects models. During this time period, the opioid crisis increased across the country. To control for the opioid epidemic, we use data on opioid overdose deaths for state and year from the Kaiser Family Foundation State Health Facts.[6] As a third set of robustness checks, we included controls for cash assistance caseloads and opioids together in the same specification. Our robustness estimates included two-way fixed effects estimates as well as estimates of increases or decreases in the policy outcome.

ETable 4 shows the robustness checks for income generosity in all three specifications: caseloads, the opioid epidemic and the two sets of variables combined. The SNAP Income Generosity variables remain negative and statistically significant for all outcomes. As a state increases the total generosity policies the child maltreatment and total foster care caseloads fall significantly. Adding in controls for other cash assistance caseloads does not significantly change the coefficients from those found in the paper; neither does controlling for the opioid epidemic or the two sets of variables combined.

© 2022 Johnson-Motoyama M et al. *JAMA Network Open.*

eReferences

1. Economic Research Service, U.S. Department of Agriculture. SNAP policy data sets. In: SNAP policy database [database online]. https://www.ers.usda.gov/data-products/snap-policy-data-sets/. Updated April 12, 2018. Accessed October 25, 2021.

2. Food and Nutrition Service, U.S. Department of Agriculture. SNAP state option reports. Cited data from 2005–2017. https://www.fns.usda.gov/snap/waivers/state-options-report. Updated August 14, 2020. Accessed October 25, 2021.

3. Food and Nutrition Service, U.S. Department of Agriculture. Office of Policy Support, Characteristics of Supplemental Nutrition Assistance Program Households. Data used from 2004-2016. https://www.fns.usda.gov/research-analysis. Accessed October 25, 2021.

4. Ganong P, Liebman JB. The decline, rebound, and further rise in SNAP enrollment: disentangling business cycle fluctuations and policy changes. Am Econ J Econ Policy. 2018;10(4):153–76.

5. Bound, J., D.A. Jaeger, and R.M. Baker. Problems with Instrumental Variables Estimation When the Correlation Between Instruments and the Endogenous Explanatory Variable is Weak. *Journal of the American Statistical Association*, 1995; 90: 443-450.

6. Kaiser Family Foundation State Health Facts. Opioid overdose deaths and opioid overdose deaths as a percent of all drug overdose deaths. https://www.kff.org/other/state-indicator/opioid-overdose-deaths. Accessed October 25, 2021.

© 2022 Johnson-Motoyama M et al. *JAMA Network Open.*

eTable 1: Data Sources and Information on Missing States/Years

SNAP Policies	Source	Information on Missing variables
Income limit as a percentage of the Federal poverty line is higher under broad-based categorical eligibility.	Snap Policy Database	
Child Support Excluded from Income Calculations	State Option Reports	Missing years imputed using value from previous year
Transitional SNAP Benefits for TANF Leavers	Snap Policy Database	
State uses the simplified reporting option that reduces requirements for changes in household circumstances.	Snap Policy Database	
**Child Maltreatment and Foster Care Variables**	**Source**	**Data Availability by Year**
Reports per 100,000 child population	NCANDS	AL, AK, GA, WI 2005-16; OR 2012-16; ND 2010-16
Children in substantiated reports per 100,000 child population	NCANDS	AL, AK, GA, WI 2005-16; OR 2012-16; ND 2010-16
Children in reports substantiated for neglect per 100,000 child population	NCANDS	AL, AK, GA, WI 2005-16; OR 2012-16; ND 2010-16
Total foster care (NCANDS) per 100,000 child population	NCANDS	AL 2009-16; AK 2006-16; GA, OR 2012-16; MI 2008-16; ND 2010-16; WI 2005-16. NC, NY & PA Missing
Total children in foster care with substantiated reports (NCANDS) per 100,000 child population	NCANDS	AL 2009-16; AK 2006-16; GA, OR 2012-16; MI 2008-16; ND 2010-16; WI 2005-16. NC, NY & PA Missing
Total children in foster care with substantiated neglect reports (NCANDS) per 100,000 child population	NCANDS	AL 2009-16; AK 2006-16; GA, OR 2012-16; MI 2008-16; ND 2010-16; WI 2005-16. NC, NY & PA Missing
Total foster care (AFCARS) per 100,000 child population	AFCARS	
Total foster care for reasons of neglect (AFCARS) per 100,000 child population	AFCARS	
**SNAP Caseload Measures**	**Source**	**Notes**
SNAP Caseloads	UKY Center for Poverty Research National Welfare Data	
SNAP Recipients	UKY Center for Poverty Research National Welfare Data	
SNAP Caseloads with Children	UKY Center for Poverty Research National Welfare Data & Characteristics of SNAP Households	Imputed by using percentages reported from Characteristics of SNAP Households
SNAP Single-Parent Family Household Caseloads	UKY Center for Poverty Research National Welfare Data & Characteristics of SNAP Households	Imputed by using percentages reported from Characteristics of SNAP Households

© 2022 Johnson-Motoyama M et al. *JAMA Network Open.*

eTable 1: Data Sources and Information on Missing States/Years (continued)

Covariates	Source
Refundable State EITC (1=Yes)	UKY Center for Poverty Research National Welfare Data
Log Real State Minimum Wage	UKY Center for Poverty Research National Welfare Data
State Uses Alternative Response	NCANDS
Screenout increased 8%	NCANDS
Screen Out Counts Missing (1 = Yes)	NCANDS
Share of Immigrants	Current Population Survey Annual Social and Economic Supplement
Unemployment Rate	UKY Center for Poverty Research National Welfare Data
Share Living in City	Current Population Survey Annual Social and Economic Supplement
Share Black-Nonhispanic	Current Population Survey Annual Social and Economic Supplement
Share Asian-Nonhispanic	Current Population Survey Annual Social and Economic Supplement
Share Other race-Nonhispanic	Current Population Survey Annual Social and Economic Supplement
Share Hispanic Any Race	Current Population Survey Annual Social and Economic Supplement
Log Real Personal Income	UKY Center for Poverty Research National Welfare Data
Share of Childen aged 3 to 4	Current Population Survey Annual Social and Economic Supplement
Share of Children aged 5 to 13	Current Population Survey Annual Social and Economic Supplement
Share of Children aged 14 to 17)	Current Population Survey Annual Social and Economic Supplement
Child Population	Current Population Survey Annual Social and Economic Supplement

© 2022 Johnson-Motoyama M et al. *JAMA Network Open.*

eTable 2: Association of Income Generosity Policies on SNAP Caseload Measures

	Log SNAP Caseloads	Log SNAP Recipients	Log SNAP Caseloads with Children	Log SNAP Caseloads with Single-Parent Family Households
State Income Generosity Policy Count	0.04***	0.05***	0.05***	0.04***
	(0.017 - 0.071)	(0.026 - 0.079)	(0.029 - 0.079)	(0.024 - 0.064)
BBCE for income	0.04	0.04	0.04	0.03
	(-0.019 - 0.092)	(-0.014 - 0.098)	(-0.016 - 0.093)	(-0.013 - 0.077)
Child Support excluded from income	0.07***	0.07***	0.08***	0.05***
	(0.031 - 0.106)	(0.034 - 0.112)	(0.035 - 0.118)	(0.024 - 0.080)
State offers transitional SNAP to leaving TANF	-0.02	0.01	0.03	0.01
	(-0.096 - 0.061)	(-0.064 - 0.081)	(-0.043 - 0.094)	(-0.048 - 0.063)
State uses simplified reporting	0.07**	0.08***	0.08***	0.11***
	(0.000 - 0.142)	(0.028 - 0.138)	(0.035 - 0.123)	(0.057 - 0.164)
Observations	663	663	663	663
Mean	10.89	10.89	10.89	10.89

SOURCE: Authors' analysis of data from University of Kentucky Center on Poverty Research and SNAP Policy Database and SNAP State Options Reports for the 50 states and District of Columbia from 2004-2016.
NOTES: *** p<.01, **p<.05, * p<.10.

© 2022 Johnson-Motoyama M et al. *JAMA Network Open.*

eTable 3: Instrumental Variables Estimates of Association of SNAP Caseloads and Child Maltreatment Outcomes and Foster Care Outcomes.

	NCANDS	NCANDS	NCANDS	NCANDS	NCANDS	NCANDS	AFCARS	AFCARS
	**Reports**	**Children in Substantiated Reports**	**Children in Substantiated Neglect Reports**	**Children in Foster Care**	**Children in Foster Care with Substantiated Reports**	**Children in Foster Care with Substantiated Neglect Reports**	**Children in Foster Care**	**Children in Foster Care for Reasons of Neglect**
Log SNAP Households with Children	-6,508.12***	-1,749.92**	-1,361.63**	-761.90***	-615.18***	-460.95**	-809.27***	-517.40**
	(-11279.03, -1737.22)	(-3148.43, -351.41)	(-2473.8, -249.47)	(-1308.71, -215.09)	(-1066.36, -164.00)	(-825.39, -96.51)	(-1381.09, -237.46)	(-938.858, -95.94)
Effect on Case Rates of 1% Increase in SNAP Caseloads	-65.08 (-112.79, -17.37)	-17.50 (-31.48, -3.51)	-13.62 (-24.74, -2.49)	-7.62 (-13.00, -2.15)	-6.15 (-10.66, -1.64)	-4.61 (-8.25, -0.97)	-8.09 (-13.81,-2.37)	-5.17 (-9.39, -0.96)
Effect on Case Rates of 5% Increase in SNAP Caseloads	-325.41 (-563.95, -86.86)	-87.50 (-157.42, -17.57)	-68.08 (-123.69, -12.47)	-38.10 (-65.44, -10.75)	-30.76 (-53.32, -8.20)	-23.05 (-41.27, -4.83)	-40.46 (-69.05, -11.87)	-25.87 (46.94, -4.80)
Mean of Dependent Variable	4289	950.7	660.8	295.2	214.6	174.5	436.4	229.9
First Stage F-Statistic	104.07	104.07	108.21	117.2	117.2	117.2	108.49	108.49
Observations	645	645	632	589	589	589	663	663

SOURCE: Authors' analysis of data from University of Kentucky Center on Poverty Research and SNAP Policy Database and SNAP State Options Reports for the 50 states and District of Columbia from 2004-2016.
NOTES: Standard errors in parentheses. *** p<.01, ** p<.05, * p<.10.

© 2022 Johnson-Motoyama M et al. *JAMA Network Open.*

eTable 4: Robustness Checks for Association of Income Generosity Policies and Child Maltreatment and Foster Care Outcomes.

	NCANDS	NCANDS	NCANDS	NCANDS	NCANDS	NCANDS	AFCARS	AFCARS
	Reports	Children in Substantiated Reports	Children in Substantiated Neglect Reports	Children in Foster Care	Children in Foster Care with Substantiated Reports	Children in Foster Care with Substantiated Neglect Reports	Children in Foster Care	Children in Foster Care for Reasons of Neglect
**Main Estimates**								
State Income Generosity Policy Count	-352.61*** (-557.068 - - 148.162)	-94.81*** (-155.577 - - 34.047)	-77.01*** (-125.427 - - 28.591)	-45.07*** (-71.637 - - 18.513)	-36.39*** (-58.136 - - 14.653)	-27.27*** (-45.077 - - 9.463)	-42.29*** (-64.781 - - 19.800)	-27.04*** (-44.290 - - 9.786)
**Controlling for Caseloads**								
State Income Generosity Policy Count	-349.13***	-106.31***	-82.34***	-41.97***	-33.83***	-24.35***	-40.22***	-26.82***
	(-522.716 - - 175.543)	(-162.020 - - 50.592)	(-127.400 - - 37.276)	(-66.850 - - 17.081)	(-52.887 - - 14.771)	(-38.828 - - 9.864)	(-61.077 - - 19.356)	(-42.756 - - 10.877)
**Controlling for Opioids**								
State Income Generosity Policy Count	-352.31***	-96.54***	-78.32***	-45.16***	-36.52***	-27.52***	-42.04***	-27.17***
	(-557.465 - - 147.155)	(-154.950 - - 38.138)	(-124.518 - - 32.118)	(-71.803 - - 18.525)	(-58.267 - - 14.778)	(-45.118 - - 9.918)	(-64.700 - - 19.379)	(-44.325 - - 10.010)
**Controlling for Caseloads & Opioids**								
State Income Generosity Policy Count	-350.18***	-109.88***	-84.87***	-42.24***	-34.10***	-24.85***	-40.26***	-27.15***
	(-524.219 - - 176.151)	(-162.628 - - 57.123)	(-127.192 - - 42.546)	(-67.183 - - 17.304)	(-53.158 - - 15.038)	(-39.234 - - 10.472)	(-61.028 - - 19.492)	(-42.853 - - 11.443)
Mean of Dependent Variable	4289	950.7	660.8	295.2	214.6	174.5	436.4	229.9
Observations	645	645	632	589	589	589	663	663

SOURCE: Authors' analysis of data from University of Kentucky Center on Poverty Research and SNAP Policy Database and SNAP State Options Reports for the 50 states and District of Columbia from 2004-2016.

© 2022 Johnson-Motoyama M et al. *JAMA Network Open.*

NOTES: Each column and row reports the effect of SNAP policies on child maltreatment outcomes from a separate regression. Robust standard errors in parentheses. *** p<.01, ** p<.05, * p<.10.

© 2022 Johnson-Motoyama M et al. *JAMA Network Open.*

eFigure 1. Percent Change in Key Indicators from 2004 to 2016, Data per 100,000 population

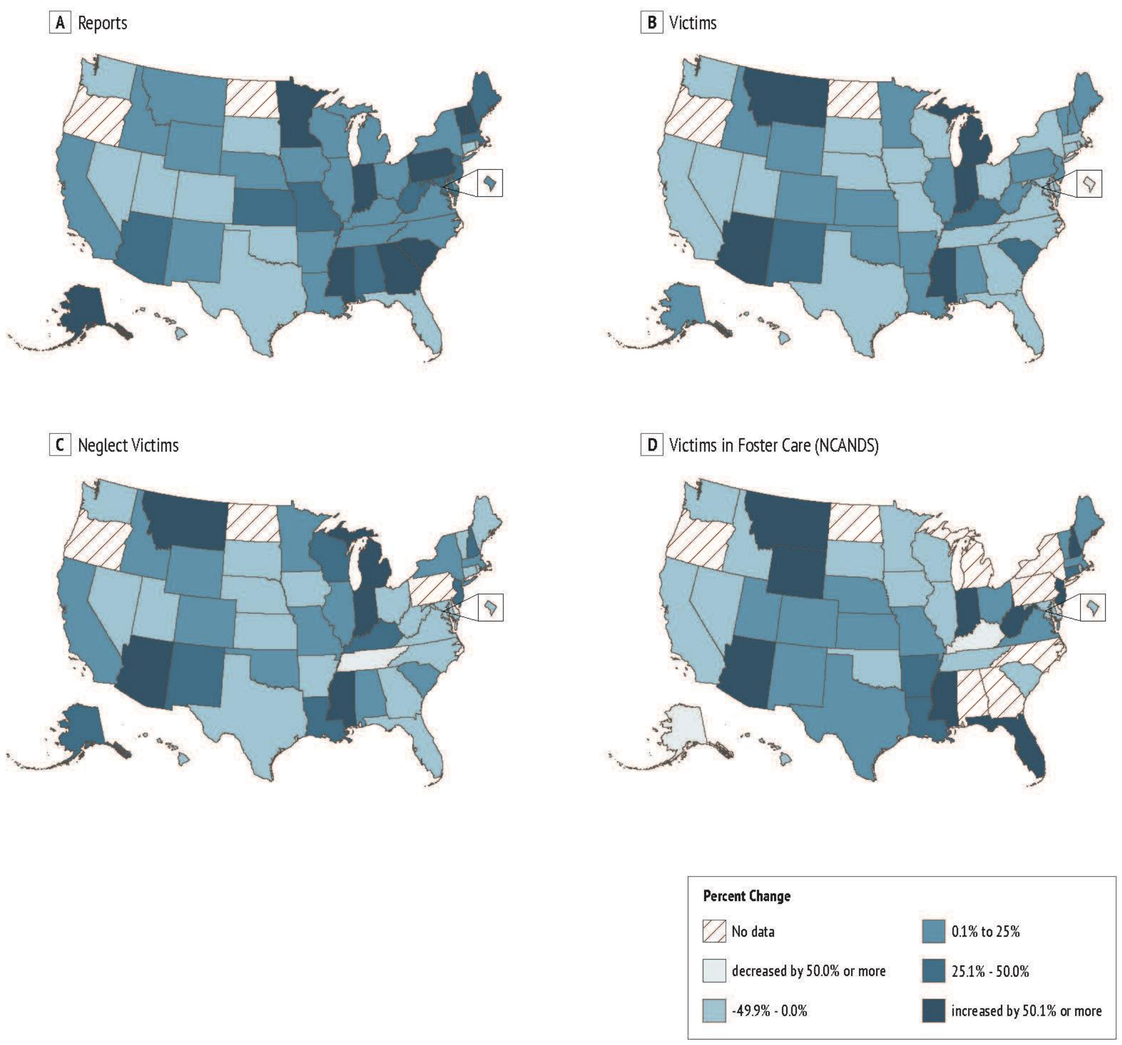

© 2022 Johnson-Motoyama M et al. *JAMA Network Open.*

eFigure 1. Percent Change in Key Indicators from 2004 to 2016, Data per 100,000 population (continued)

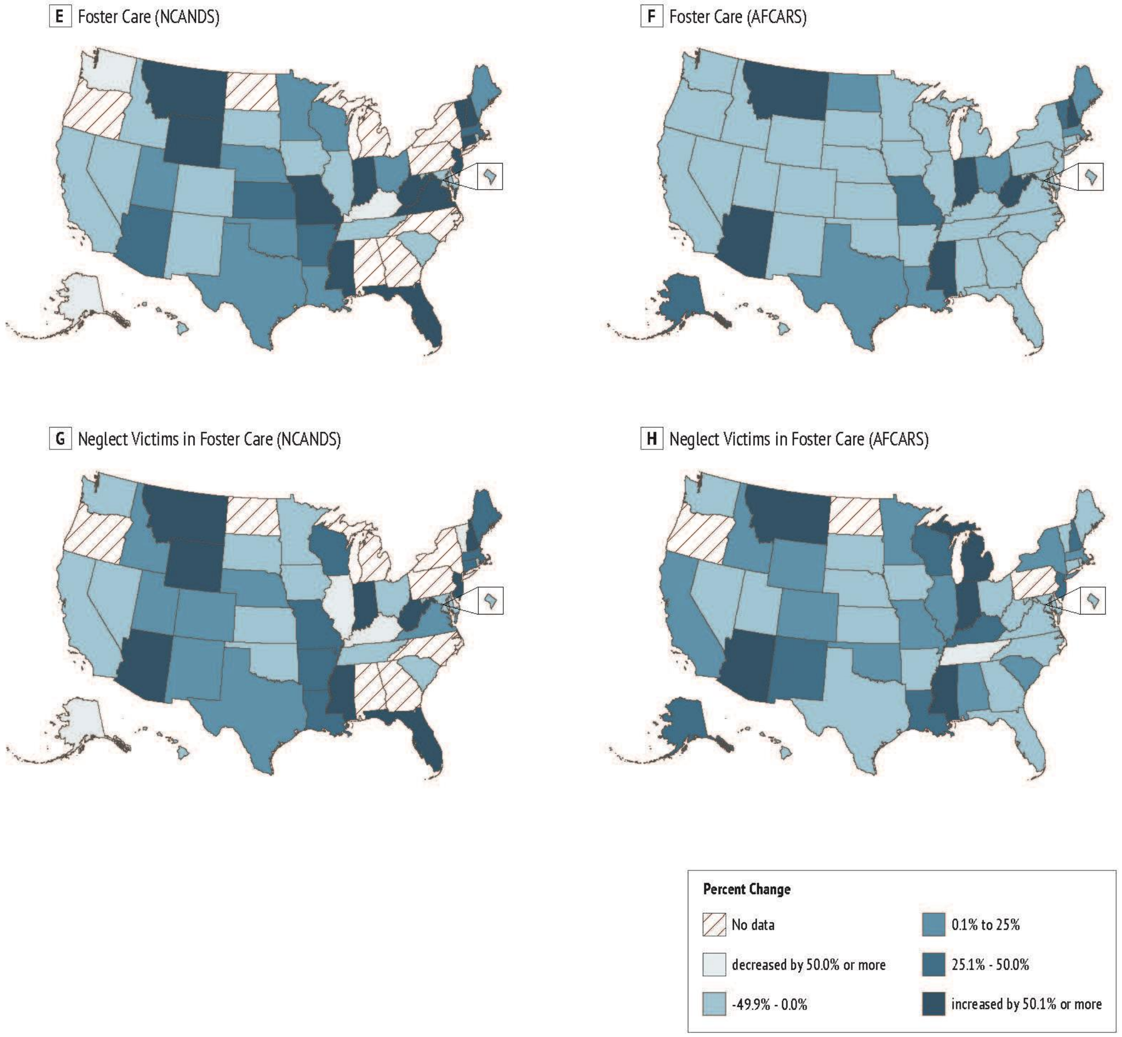

Source: Institute for Policy & Social Research, The University of Kansas; data from Economic Research Service (ERS), U.S. Department of Agriculture (USDA). SNAP Policy Database, SNAP Policy Data Sets. https://www.ers.usda.gov/data-products/snap-policy-data-sets/

© 2022 Johnson-Motoyama M et al. *JAMA Network Open.*

eFigure 2. Association of Child Maltreatment and Foster Care Case Rates per 100,000 Population and SNAP Income Policies– Comparing Fixed Effects and Instrumental Variables Estimates

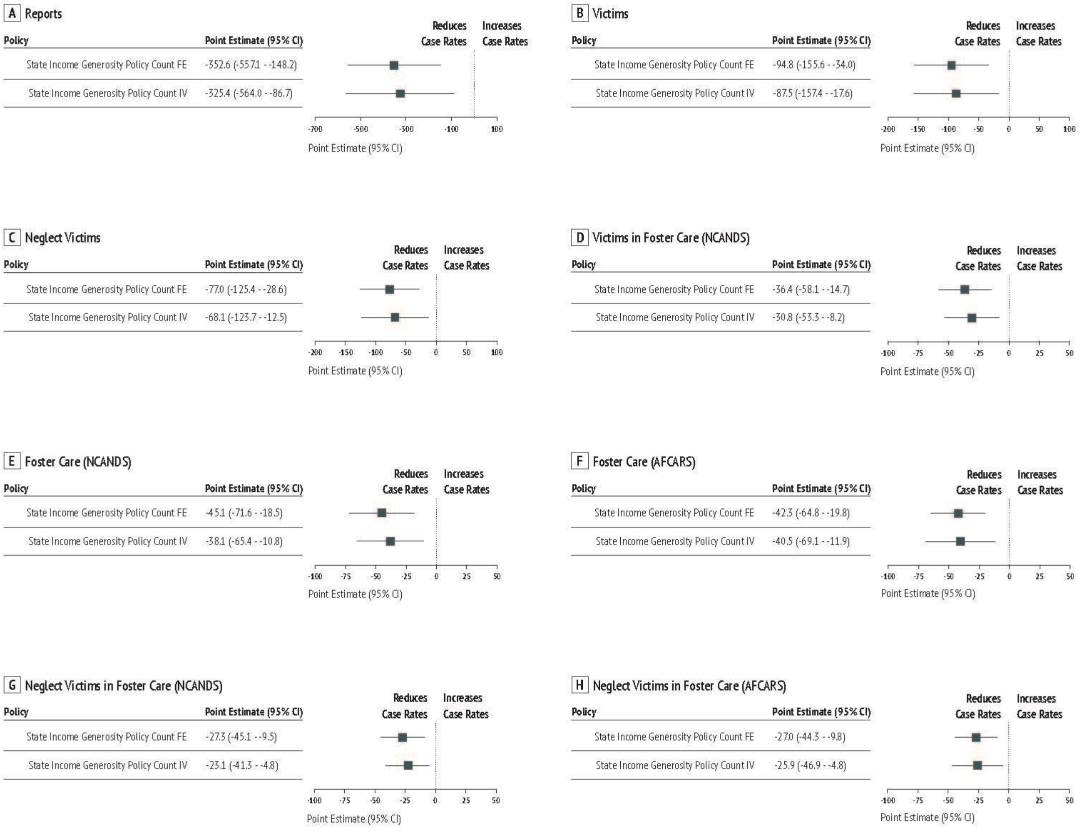

© 2022 Johnson-Motoyama M et al. *JAMA Network Open.*
